# Molecular detection and identification of tick-borne bacteria and protozoans in goats and wild Siberian roe deer (*Capreolus pygargus*) from Heilongjiang Province, northeastern China

**DOI:** 10.1186/s13071-019-3553-1

**Published:** 2019-06-13

**Authors:** Haoning Wang, Jifei Yang, Muhammad Uzair Mukhtar, Zhijie Liu, Minghai Zhang, Xiaolong Wang

**Affiliations:** 1 College of Wildlife Resource, Northeast Forestry University, Harbin, 150040 Heilongjiang People’s Republic of China; 2 State Key Laboratory of Veterinary Etiological Biology, Key Laboratory of Veterinary Parasitology of Gansu Province, Lanzhou Veterinary Research Institute, Chinese Academy of Agricultural Sciences, Xujiaping 1, Lanzhou, 730046 Gansu People’s Republic of China; 3 Center of Conservation Medicine & Ecological Safety, Northeast Forestry University, Harbin, 150040 Heilongjiang People’s Republic of China

**Keywords:** Tick-borne pathogen, *Theileria*, *Anaplasma*, Zoonosis, Goats, Siberian roe deer, China

## Abstract

**Background:**

Small ruminants are important hosts for various tick species and tick-associated organisms, many of which are zoonotic. The aim of the present study was to determine the presence of tick-borne protozoans and bacteria of public health and veterinary significance in goats and wild Siberian roe deer (*Capreolus pygargus*) from Heilongjiang Province, northeastern China.

**Methods:**

The occurrence of piroplasms, *Anaplasma phagocytophilum*, *A. bovis*, *A. marginale*, *A. capra*, *A. ovis*, *Ehrlichia* spp. and spotted fever group rickettsiae was molecularly investigated and analyzed in 134 goats and 9 free ranging *C. pygargus* living in close proximity.

**Results:**

Piroplasm DNA was detected in 16 (11.9%) goats and 5 *C. pygargus*. Sequence analysis of *18S* rRNA sequences identified 3 *Theileria* species (*T. luwenshuni*, *T. capreoli* and *T. cervi*). Four *Anaplasma* species (*A. ovis*, *A. phagocytophilum*, *A. bovis* and *A. capra*) were identified in goats and *C. pygargus*. *Anaplasma ovis* and *A. bovis* were detected in 11 (8.2%) and 6 (4.5%) goats, respectively; *A. phagocytophilum*, *A. bovis* and *A. capra* were found in 3, 7 and 3 *C. pygargus*, respectively. Sequence analysis of *16S* rRNA sequences revealed the presence of 5 different genetic variants of *A. bovis* in goats and *C. pygargus*, while the analysis of *16S* rRNA and *gltA* sequence data showed that *A. capra* isolates identified from *C. pygargus* were closely related to the genotype identified from sheep and *Haemaphysalis qinghaiensis*, but differed with the genotype from humans. *Anaplasma*/*Theileria* mixed infection was observed in 2 (1.5%) goats and 5 *C. pygargus*, and co-existence involving potential zoonotic organisms (*A. phagocytophilum* and *A. capra*) was found in 2 *C. pygargus*. All samples were negative for *A. marginale*, *Ehrlichia* spp. and SFG rickettsiae.

**Conclusions:**

These findings report the tick-borne pathogens in goats and *C. pygargus*, and a greater diversity of these pathogens were observed in wild animals. Three *Theileria* (*T. luwenshuni*, *T. capreoli* and *T. cervi*) and four *Anaplasma* species (*A. ovis*, *A. phagocytophilum*, *A. bovis* and *A. capra*) with veterinary and medical significance were identified in small domestic and wild ruminants. The contact between wild and domestic animals may increase the potential risk of spread and transmission of tick-borne diseases.

## Background

Small ruminants are known to harbor various ticks that act as vectors and reservoirs of tick-borne pathogens of veterinary and/or medical importance. They play an important role, not only in the life-cycle of ticks, but also in the natural maintenance and transmission of these pathogens [1]. Tick-borne diseases affect domestic and wild ruminants, especially sheep, goats, cattle and deer [2]. In the past two decades, an increasing number of tick-borne pathogens have been identified in domestic animals and a variety of wild cervids, some of which have been confirmed as causes of human infection, such as *Anaplasma phagocytophilum*, *Anaplasma capra*, *Babesia divergens*, *Babesia venatorum*, *Ehrlichia canis*, etc. [3–6]. This fact is of great concern in terms of tick-borne disease control, since animals are usually asymptomatic carriers that may serve as reservoirs of the infection.

With advances in molecular techniques, a number of well-known and potential novel tick-borne bacteria and protozoans have been identified in unexpected hosts and geographical locations [7]. In China, *Theileria annulata* is the causative agent of bovine theileriosis and has been identified in sika deer [8]; *Theileria uilenbergi* causes ovine theileriosis and has been detected in red deer and sika deer [9]; and *Babesia motasi* infects sheep and goats and has been reported in sika deer [8]. This information warrants further investigation of tick-borne pathogens in both domestic and wild animals simultaneously. Siberian roe deer (*Capreolus pygargus*) is a commonly encountered wild animal and distributed mainly in Xinjiang and northeastern China [10]. The aim of this study was to determine the occurrence of the tick-borne bacteria and protozoans in goats and free-ranging *C. pygargus* from Heilongjiang Province, northeastern China.

## Methods

### Study sites and collection of specimens

In the present study, EDTA-anticoagulated blood samples were collected from 134 asymptomatic goats and 9 free-ranging *C. pygargus* from September 2017 to August 2018 in forest farms in Hebei Forestry Bureau, Mudanjiang city and Chaihe Forestry Bureau from Heilongjiang Province, northeastern China. The forest farms are local administrative units which have changed their role from timber production to forest and wildlife conservation over the last two decades. They never serve as large scale husbandry units, while backyard livestock breeding by its employees is common. Those domestic animals graze freely and share a common habitat with wild ungulates, which can frequently be found feeding very close to the local settlements. The samples were collected from rescued *C. pygargus* during the course of daily routine patrol by local wildlife disease monitoring stations. The goats were sampled in the herds close to the sites where *C. pygargus* included in this study were found. Sample collection and animal handling complied with the Animal Ethics Procedures and Guidelines and was approved by the Animal Ethics Committee of Northeast Forestry University. Genomic DNA was extracted from 200 μl of whole blood using a QIAamp DNA Mini Kit (Qiagen, Hilden, Germany) according to the instructions of the manufacturer.

### PCR reactions

The extracted DNA was screened for the presence of piroplasms, *Anaplasma*, *Ehrlichia* and spotted fever group (SFG) rickettsiae by PCR. The PCR primers and cycling conditions used in this study are listed in Table 1. Briefly, nested PCRs were employed for the detection of piroplasms, *A. phagocytophilum*, *A. bovis*, and *A. capra* based on *18S* rRNA, *16S* rRNA and *gltA* genes, respectively. *Anaplasma ovis*, *A. marginale*, *Ehrlichia* spp. and SFG rickettsiae were detected by conventional PCR based on *msp4* gene, *16S* rRNA and *ompA* genes, respectively. PCR reactions were conducted in an automatic thermocycler (Bio-Rad, Hercules, CA, USA) in a total volume of 25 μl, including 2 μl of DNA sample as previously described [11]. The DNAs extracted from the whole blood of animals infected with *T. annulata*, *A. phagocytophilum*, *A. bovis*, *A. marginale* and *A. ovis*, and the DNAs from ticks positive for *E. chaffeensis* and SFG rickettsiae that had been verified by sequencing, were used as the positive control for corresponding PCR reactions; sterile water was used as the blank control for each run. Amplified fragments were electrophoresed on a 1.0% agarose gel containing 10 μl of GoldView (SolarBio, Beijing, China) and visualized under UV transillumination.

**Table 1:** Primers and PCR amplification conditions

Pathogen	Target gene	Primer name	Primer sequence (5′–3′)	Annealing T (°C)	Amplicon size (bp)	References
Piroplasm	*18S* rRNA	Piro1-S	CTTGACGGTAGGGTATTGGC	55	~ 1410	[24, 25]
		Piro3-AS	CCTTCCTTTAAGTGATAAGGTTCAC			
		PIRO-A1	CGCAAATTACCCAATCCTGACA	55	~ 430	
		PIRO-B	TTAAATACGAATGCCCCCAAC			
*A. phagocytophilum*	*16S* rRNA	EE1	CCTGGCTCAGAACGAACGCTGGCGGC	55	~ 1430	[26, 27]
		EE2	AGTCACTGACCCAACCTTAAATGGCTG			
		SSAP2f	GCTGAATGTGGGGATAATTTAT	60	641	
		SSAP2r	ATGGCTGCTTCCTTTCGGTTA			
*A. bovis*	*16S* rRNA	EE1	TCCTGGCTCAGAACGAACGCTGGCGGC	55	~ 1430	[26, 27]
		EE2	AGTCACTGACCCAACCTTAAATGGCTG			
		AB1f	CTCGTAGCTTGCTATGAGAAC	60	551	
		AB1r	TCTCCCGGACTCCAGTCTG			
*A. marginale*	*msp4*	AmargMSP4Fw	CTGAAGGGGGAGTAATGGG	60	344	[28]
		AmargMSP4Rev	GGTAATAGCTGCCAGAGATTCC			
*A. ovis*	*msp4*	MSP45	GGGAGCTCCTATGAATTACAGAGAATTGTTTAC	55	869	[29]
		MSP43	CCGGATCCTTAGCTGAACAGAATCTTGC			
*A. capra*	*gltA*	Outer-f	GCGATTTTAGAGTGYGGAGATTG	55	1031	[6]
		Outer-r	TACAATACCGGAGTAAAAGTCAA			
		Inner-f	TCATCTCCTGTTGCACGGTGCCC	60	594	[22]
		Inner-r	CTCTGAATGAACATGCCCACCCT			
	*16S* rRNA	Forward	GCAAGTCGAACGGACCAAATCTGT	58	1261	[30]
		Reverse	CCACGATTACTAGCGATTCCGACTTC			
*Ehrlichia* spp.	*16S* rRNA	ECC	AGAACGAACGCTGGCGGCAAGC	60	450	[26]
		ECB	CGTATTACCGCGGCTGCTGGCA			
SFG rickettsiae	*OmpA*	Rr190.70	ATGGCGAATATTTCTCCAAAA	55	632	[31]
		Rr190.701	GTTCCGTTAATGGCAGCATCT			

Abbreviation: T, temperature

### DNA sequencing and phylogenetic analysis

The DNA fragments were purified with a AxyPrepTM DNA Gel Extraction Kit (Axygen, Union City, CA, USA), cloned into pGEM-T Easy vector (Promega, Madison, WI, USA) and transformed for sequencing using BigDye Terminator Mix (GenScript, Nanjing, China). The nucleotide sequences obtained in this study were compared with previously published sequences deposited in GenBank by a BLASTn search or by using the ClustalW multiple alignment algorithm in the MegAlign program of the Lasergene 7.1 software package (DNAStar, Madison, WI, USA). The phylogenetic trees were inferred by using the neighbor-joining (NJ) method with the Kimura two-parameter model, and the bootstrap test was replicated 1000 times [12].

### Nucleotide sequence accession numbers

The representative sequences of the identified pathogens in this study were deposited in the GenBank database and assigned accession numbers as follows: MH085202, MH085203 and MK271372 for *18S* rRNA gene sequences of *T. capreoli*, *T. cervi* and *T. luwenshuni*, respectively; MH085195–MH085196 and MK271373–MK271375 for *16S* rRNA gene sequences of *A. bovis*; MH085197 and MH085198 for *16S* rRNA gene sequences of *A*. *phagocytophilum* and *A. capra*, respectively; MK271379 for the *msp4* gene sequence of *A. ovis*; and MH094751 for the *gltA* gene sequence of *A. capra*.

## Results

**Fig. 1:**
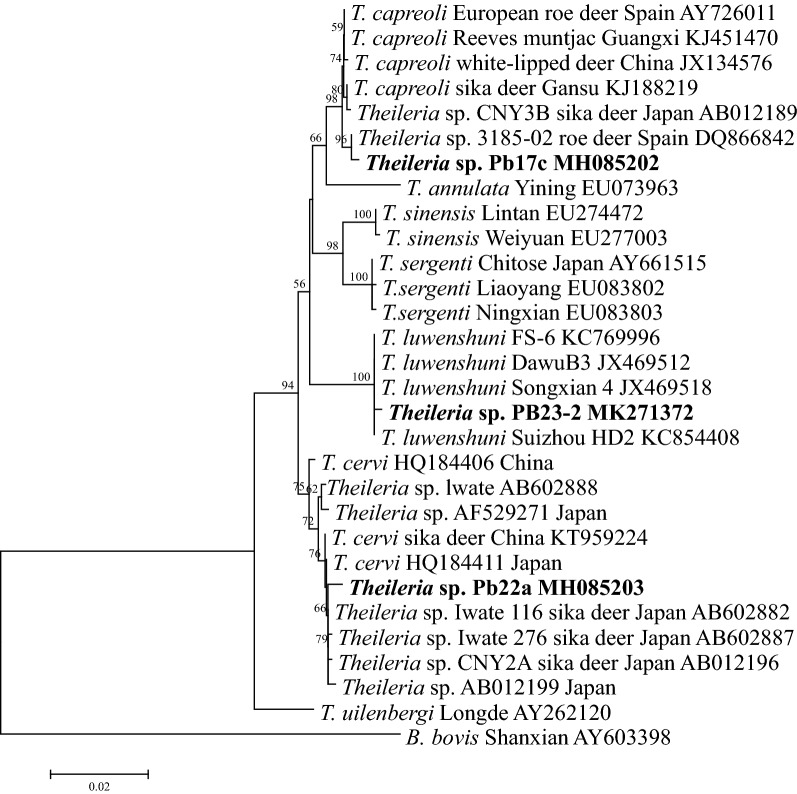
Phylogenetic analysis of the *Theileria* species identified in this study based on the *18S* rRNA gene. *Babesia bovis* was used as outgroup. Boldface indicates the sequences obtained in this study. The phylogenetic trees were inferred by using the neighbor-joining (NJ) method with the Kimura two-parameter model, and the bootstrap test was replicated 1000 times. There was a total of 1367 positions in the final dataset

Sixteen (11.9%) of 134 goats and 5 (55.6%) of 9 free-ranging *C. pygargus* tested positive for piroplasms by nested PCR, which amplifies an approximately 430 bp band of the *18S* rRNA gene of *Theileria*/*Babesia* spp. All amplicons were sequenced, and BLAST analysis revealed that the obtained sequences belonged to three different *Theileria* species; *Babesia* was not identified in any of these amplicon sequences. Sequence analysis revealed that the *18S* rRNA sequences detected from goats were 100% identical to each other and to the isolates GNhl4 (MG799814), SX01 (MG930123) and PZG5 (LC326009) of *T. luwenshuni* identified in *Haemaphysalis longicornis* and goats from China and Myanmar. The *18S* rRNA sequences of *Theileria* detected from *C. pygargus* were classified into two groups. Two *18S* rRNA sequences were 100% identical to the *Theileria* sp. 3185/02 (GenBank: DQ866842) identified in roe deer (*Capreolus capreolus*) from Spain and the isolate TCCRO1 of *T. capreoli* (GenBank: KY359359) identified in grey wolf (*Canis lupus*) from Croatia [13]. Three sequences were 100% identical to the isolates Am4 (GenBank: MG041373) and 13WYs1a (GenBank: KP407020) of *T. cervi* isolated from *Ixodes persulcatus* and sika deer (*Cervus nippon*) in Russia and China, respectively. To further characterize these three *Theileria* species, the first-round PCR amplicons from positive samples (~ 1410 bp) were sequenced. Sequence and phylogenetic analysis revealed that *Theileria* sp. (PB23-2, GenBank: MK271372) identified from goats was closely related to *T. luwenshuni* identified from sheep and goats (GenBank: KC769996, JX469512, JX469518 and KC854408) in China; *Theileria* sp. (Pb17c, GenBank: MH085202) identified from *C. pygargus* was clustered together with *T. capreoli* isolates from roe deer (GenBank: AY726011 and DQ866842) in Spain, Reeves’ muntjac (GenBank: KJ451470) and white-lipped deer (GenBank: JX134576) in China and sika deer in Japan (GenBank: AB012189); *Theileria* sp. (Pb22a, GenBank: MH085203) from *C. pygargus* was closely related to *T. cervi* identified from sika deer in China and Japan (GenBank: HQ184411, KT959224, AB602882, AB602887, AB012196 and AB012199) (Fig. 1).

Four *Anaplasma* species were detected in goats and *C. pygargus*, including *A. ovis*, *A. bovis*, *A. phagocytophilum* and *A. capra*. *Anaplasma ovis* was detected in 11 (8.2%) of 134 goats. The *msp4* sequences (MK271379) detected in goats were 100% identical to the *A. ovis* isolates from sheep (GenBank: MF071307 and AY702924) in China and Italy. *Anaplasma bovis* infection was found in 6 (4.5%) goats and 7 (77.8%) *C. pygargus*. The *16S* rRNA sequences (551 bp) amplified from goats and *C. pygargus* provided five sequence variants that had 98.8–99.8% identity. These *A. bovis* variants (Ab27-2, Ab35-1 and Ap45-3 from goats, MK271373–MK271375; Ab18a and Ab21b from *C. pygargus*, MH085195 and MH085196) were 99.3–100% identical to the *A. bovis* isolate b2-25a (GenBank: MF066914) from sheep in China. *Anaplasma phagocytophilum* was detected in three (33.3%) *C. pygargus*. The *16S* rRNA sequences of *A. phagocytophilum* (641 bp) obtained from this study (Ap23a, MH085197) were 100% identical to each other and to isolate ApGOv1 derived from sheep (GenBank: KM285230) in Tunisia, GS29 from cattle (GenBank: GU223365) in Turkey, and Ac30B from sika deer (GenBank: AB588976) in Japan.

**Fig. 2:**
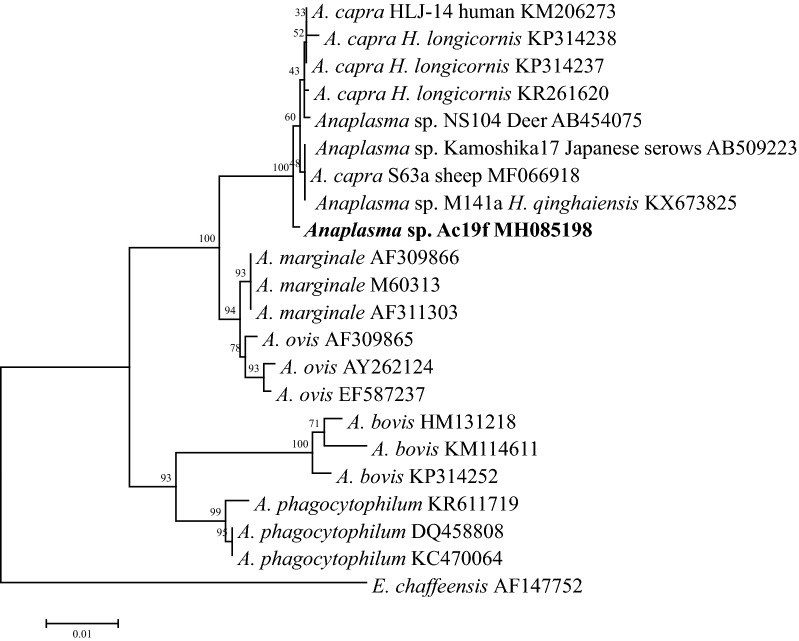
Phylogenetic analysis of the *Anaplasma capra* based on the *16S* rRNA gene. *Ehrlichia chaffeensis* was used as outgroup. Boldface indicates the sequences obtained in this study. The phylogenetic trees were inferred by using the neighbor-joining (NJ) method with the Kimura two-parameter model, and the bootstrap test was replicated 1000 times. There was a total of 1219 positions in the final dataset

**Fig. 3:**
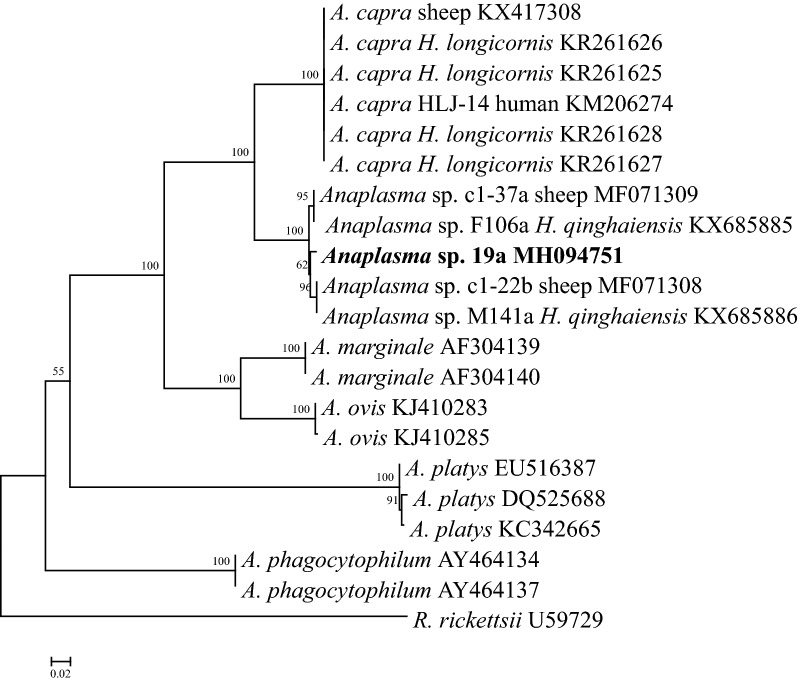
Phylogenetic analysis of the *Anaplasma capra* based on the *gltA* gene. *Rickettsia rickettsii* was used as outgroup. Boldface indicates the sequences obtained in this study. The phylogenetic trees were inferred by using the neighbor-joining (NJ) method with the Kimura two-parameter model, and the bootstrap test was replicated 1000 times. There was a total of 563 positions in the final dataset

Three *C. pygargus* (33.3%) were positive for *A. capra*. These *A. capra* isolates were molecularly characterized based on *16S* rRNA and *gltA* genes. The *16S* rRNA gene sequences (Ac19f, MH085198) were 99.8% identical to the *A. capra* isolate S63a identified from sheep (MF066918), *Anaplasma* sp. Kamoshika17 from Japanese serows (GenBank: AB509223), *Anaplasma* sp. NS104 from deer (GenBank: AB454075), M141a from *H. qinghaiensis* ticks (GenBank: KX673825), and 99.7% identical to the isolate HLJ-14 of *A. capra* (GenBank: KM206273) isolated in humans (GenBank: KM206274). The *gltA* sequences (Ac19a, MH094751) were 98.5–98.7% identical to *A. capra* isolates from sheep and *H. qinghaiensis* ticks (GenBank: MF071308, MF071309, KX685885 and KX685886), but they had a low sequence identity (87.6%) to the corresponding sequence of *A. capra* HLJ-14 from humans (GenBank: KM206274). Phylogenetic analyses revealed that the isolate identified in *C. pygargus* was clustered into the *A. capra* clade based on *16S* rRNA gene, distinct from other well-recognized *Anaplasma* species (Fig. 2). However, based on the *gltA* gene, the isolate was closely related to *A. capra* strains from sheep and *H. qinghaiensis*, but separated clearly from the human isolate HLJ-14 (Fig. 3).

Co-infection with *Anaplasma* and *Theileria* was observed in 2 goats and 5 *C. pygargus*. Co-infection that involved potential zoonotic organisms (*A. phagocytophilum* and *A. capra*) was found in 2 *C. pygargus*. Moreover, all animals included in this study tested negative for *A. marginale*, *Ehrlichia* spp. and SFG rickettsiae.

## Discussion

The occurrence of tick-borne pathogens has been well documented in various domestic and wild ruminants in many countries. In addition to their veterinary importance, many tick-borne pathogens are known to have zoonotic potential. In the present study, the presence of tick-borne bacteria and protozoans was investigated in domestic and wild small ruminants; three *Theileria* (*T. luwenshuni*, *T. capreoli* and *T. cervi*) and four *Anaplasma* species (*A. ovis*, *A. phagocytophilum*, *A. bovis* and *A. capra*) were identified in goats and free-ranging *C. pygargus*.

Protozoan parasites of the genus *Theileria* are obligatory intracellular parasites that infect leukocytes and erythrocytes of both wild and domestic animals. Aside from highly pathogenic *Theileria* species, such as *T. parva* and *T. annulata*, others are described as low pathogenic [13]. Asymptomatic infections by different *Theileria* species have so far been identified in a variety of wild and domestic animals [1, 14–17]. In this study, *T. luwenshuni* was identified in goats, and *T. capreoli* and *T. cervi* in *C. pygargus* in Heilongjiang Province, northeastern China. *Theileria luwenshuni* is a new *Theileria* species highly pathogenic for small domestic ruminants (goats and sheep); it is widely distributed in China and causes substantial economic losses for the livestock industry [18]. *Theileria capreoli* and *T. cervi* have been frequently reported in cervid species. *Theileria capreoli* was first described in roe deer, and recorded subsequently in red deer, fallow deer, roe deer and Chinese water deer [16, 19]. *Theileria cervi* is a non-pathogenic species that has been reported in brown brocket deer, white-tailed deer, sika deer, axis deer, marsh deer, elk, pampas deer and mule deer [14, 15, 20]. Our findings suggest that *C. pygargus* may serve as a reservoir of *T. capreoli* and *T. cervi* in northeastern China.

The genus *Anaplasma* encompasses a group of obligate intracellular bacteria that are causative agents of anaplasmosis with veterinary and public health significance [5]. They have different cellular tropism, vectors, pathogenicity and host range. In this study, *A. ovis* and *A. bovis* were identified in goats, and *A. phagocytophilum* and *A. bovis* in free-ranging *C. pygargus*. *Anaplasma ovis*, *A. phagocytophilum* and *A. bovis* are frequently detected tick-borne pathogens in small ruminants around the world; however, the infection of *A. phagocytophilum* was not found in goats included in this study. Aside from *A. ovis*, *A. phagocytophilum* and *A. bovis* have the broadest host range. Our results suggested that the *C. pygargus* may serve as reservoir hosts for *A. phagocytophilum* and *A. bovis*. Considering the fact that *A. phagocytophilum* and *A. bovis* can affect a variety of domestic and wild animals, the risk of cross-infection is high in areas where wild and domestic animals share a common habitat.

Apart from those well-known *Anaplasma* species, a novel *Anaplasma* species named *A. capra* has been recently described in goats and identified subsequently as a human pathogen in Heilongjiang Province in northeastern China [6]. The molecular investigations of *A. capra* in small domestic ruminants and several tick species revealed that this pathogen is widely distributed in China, subdivided into two genotypes [11, 21, 22]. In this study, *A. capra* was identified in *C. pygargus* in Heilongjiang Province, where the human isolate HLJ-14 was described [6]. Phylogenetic analysis showed that the isolates from *C. pygargus* were closely related to *A. capra* genotype identified from sheep and *H. qinghaiensis*, but differ with the genotype from human, suggesting the high degree of genetic diversity of this agent. Moreover, since *16S* rRNA sequences of *A. capra* have been previously detected in Japanese serows (AB509223) and deer (*Anaplasma* sp. NS104, AB454075) from Japan [23], the finding of this agent in *C. pygargus* was not surprising. These findings, together with previous reports, suggest that *A. capra* could be efficiently maintained in nature through enzootic cycles between ticks and wild cervids, and the *A. capra* genotype identified in *C. pygargus* is distinct from the human genotype.

In summary, several tick-borne pathogens were identified in small domestic and wild ruminants from northeastern China; three (*T. luwenshuni*, *A. ovis* and *A. bovis*) were identified in goats and five (*T. capreoli*, *T. cervi*, *A. phagocytophilum*, *A. bovis* and *A. capra*) in free-ranging *C. pygargus*. These findings suggest a greater diversity of tick-borne pathogens in wild animals than that in domestic animals. Furthermore, the infection of *A. marginale*, *Ehrlichia* spp. and SFG rickettsiae was not found in those animals included this study. Goats and *C. pygargus* serve as reservoirs for tick-borne protozoans and bacteria with a broad host range, such as *A. phagocytophilum*, *A. bovis* and *A. capra*, posing a potential threat to domestic and other wild animals as well as humans.

## Conclusions

This study describes the occurrence of tick-borne bacteria and protozoans in goats and *C. pygargus* from northeastern China. Three *Theileria* (*T. luwenshuni*, *T. capreoli* and *T. cervi*) and four *Anaplasma* species (*A. ovis*, *A. phagocytophilum*, *A. bovis* and *A. capra*) with veterinary and medical significance were identified in small domestic and wild ruminants. The identification of these causative agents in domestic and wild animals provides useful information for the control and management of tick-borne diseases.

## Data Availability

Sequences have been submitted in the GenBank database under the following Accession Numbers: MH085202, MH085203 and MK271372 for *18S* rRNA gene sequences of *T. capreoli*, *T. cervi* and *T. luwenshuni*, respectively; MH085195–MH085196 and MK271373–MK271375 for *16S* rRNA gene sequences of *A. bovis*; MH085197 and MH085198 for *16S* rRNA gene sequences of *A*. *phagocytophilum* and *A. capra*, respectively; MK271379 for the *msp4* gene sequence of *A. ovis*; and MH094751 for the *gltA* gene sequence of *A. capra*.

## Abbreviations

PCR: polymerase chain reaction
DNA: deoxyribonucleic acid
*gltA*: the citrate synthase gene
*msp*: major surface protein
UV: ultraviolet
